# Next-generation leadership development in family businesses: the critical roles of shared vision and family climate

**DOI:** 10.3389/fpsyg.2014.01335

**Published:** 2014-12-04

**Authors:** Stephen P. Miller

**Affiliations:** Family Enterprise Center, UNC Kenan-Flagler Business SchoolChapel Hill, NC, USA

**Keywords:** family business, family climate, intergenerational authority, leadership effectiveness, next-generation leader, open communication, shared vision, work engagement

## Abstract

The multigenerational survival rate for family-owned businesses is not good. Lack of a shared vision for the family enterprise and weak next-generation leadership are often cited as two of the leading reasons for the failure of family firms to successfully transition from one generation of family ownership to the next. The climate of the business-owning family has also been suggested as important to the performance of the family enterprise. Despite these commonly held tenets, there is a lack of rigorous quantitative research that explores the relationships among these three factors. To address this gap, a quantitative study of 100 next-generation family firm leaders and 350 family and non-family leaders and employees with whom they work was conducted. The results demonstrate that a shared vision for the family business has a strong effect on the leadership effectiveness of next-generation family leaders and a moderate effect on the degree to which they are positively engaged with their work. The findings also show that two dimensions of family climate significantly influence the likelihood that a shared vision for the family firm has been created. Open communication in the family is positively related to the presence of a shared vision for the business. Intergenerational authority, which refers to a senior generation that exercises unquestioned authority and sets the rules, is negatively related to the presence of a shared vision. Surprisingly, a third dimension of family climate, cognitive cohesion, which includes shared values in the family, had no relationship with the degree to which there was a shared vision for the family business. The implications for family business owners is that they would be wise to spend as much time on fostering a positive family climate characterized by open communication as they do on creating and executing a successful business strategy if their goal is to pass the business from one generation of family owners to the next.

## Introduction

Family businesses constitute between 80 and 98% of all businesses in the world's free economies, generate 49% of the GDP in the U.S. and more than 75% in most other countries. They employ 80% of the U.S. workforce and more than 75% of the working population globally, and created 86% of all new jobs in the U.S. over the past decade. Despite their importance, only 30% of family businesses survive from the first to the second generation of family ownership, only 12% survive from the second generation to the third, and only 4% survive from the third generation to the fourth (Poza, 2013).

A survey of family business owners conducted by Ward (1997) found that lack of a shared vision for the family firm and weak next-generation leadership were two of the top three threats to long-term family firm success. While shared vision is important to the vitality of any business, it is of critical importance to family enterprises as commitment of family owners to the vision for the business is necessary to ensure long-term survival (Carlock and Ward, 2001; Ward, 2004; Poza, 2013). Well prepared next-generation family leaders who are committed to the vision of the family firm and engaged with their work are critical to smooth leadership successions (Handler, 1992, 1994; Morris et al., 1997; Sharma and Irving, 2005). Research has also shown that the climate of a business-owning family plays an important role in determining the culture and performance of family firms (Dyer, 1986; Björnberg and Nicholson, 2007) and the likelihood of successful transitions from one generation to the next (Morris et al., 1997).

This study explores the relationships among shared vision, family climate, and next-generation leadership effectiveness and engagement with their work in the family firm. There is a lack of empirical quantitative research to help us understand if these factors that are so often linked with family business success and longevity are actually related to each other. This paper seeks to address that gap in the literature, although it makes no attempt to demonstrate their relationship with long-term family firm success, a task that would be more adequately addressed by a longitudinal study. Three overarching questions motivate the research. Does family climate affect the development of a shared vision for the family enterprise? Does shared vision predict the effectiveness of next-generation family firm leaders? Is a shared vision related to the degree to which next-generation family leaders are positively engaged with their work in the family business?

A quantitative study informed by leadership and family systems theories was designed to answer these questions. The sample for the study included 100 next-generation family leaders of privately-owned family businesses and 350 family and non-family members of their firms familiar with their leadership behaviors. The results supported hypothesized relationships between two dimensions of family climate and shared vision, but contradicted the expected outcome for a third. Shared vision turned out to be a strong predictor of next-generation leadership effectiveness, with a smaller but still significant impact on the degree to which next-generation family leaders were engaged with their work. While the survey design and correlational method employed for the study cannot provide enough evidence to support a definitive conclusion because the underlying mechanism is not known, the results support the idea that shared vision serves as a mediator through which family climate influences next-generation leadership effectiveness and engagement with work.

The paper is organized as follows. First, a brief review of key theories that informed the development of hypotheses and a conceptual structural equation model is provided. A detailed description of the research methods and results of data analysis follow. The paper concludes with a discussion that includes interpretations of results, implications for family business practice, suggestions for future research, and limitations of the study.

### Leadership effectiveness

Leadership is a complex and multi-dimensional concept. In his meta-analysis of leadership studies, Wren (2006) identified no less than 53 approaches to leadership research, all with their own nuanced definitions of effective leadership. Emotional and social intelligence, full-range leadership, authentic leadership, and leader-member exchange are several of the leading contemporary theories of leadership.

Emotional and social intelligence refers to leadership behaviors that reflect self-awareness, self-management, social awareness, and relationship management (Goleman et al., 2002). Studies have shown that as much as 90% of a leader's effectiveness is determined by his/her emotional and social intelligence (Cherniss and Adler, 2000).

Full-range leadership theory includes transformational, transactional, and laissez-faire leadership (Bass, 1985; Antonakis et al., 2003). Transformational leadership inspires followers through charisma; a strong commitment to values, beliefs, and mission; the ability to communicate an inspirational vision of the future; intellectual stimulation; and individualized attention to the interests and needs of followers. Transactional leadership motivates follower compliance through promises, praise, and/or rewards; and corrects non-compliance with negative feedback, reproof, threats, and/or disciplinary actions (Bass and Steidlmeier, 1999). Laissez-faire leadership refers to a leader's “active” choice to avoid responsibility, decision-making, and the exercise of authority (Antonakis et al., 2003). While situational in nature, transformational leadership has been found to be generally more effective than transactional leadership, with laissez-faire leadership the least effective of the three (Antonakis et al., 2003).

Bass and Steidlmeier (1999) add a moral dimension to full-range leadership characteristics in defining “authentic leadership,” which seeks to differentiate charismatic leaders who produce positive results for the organizations they lead from those who use the same characteristics to manipulate followers for their own selfish ambitions. Avolio et al. (2009) provide a more comprehensive definition of authentic leadership that includes objectively reviewing relevant data and considering multiple perspectives before making a decision, self-regulated behavior guided by an internal moral compass, relational transparency characterized by open communication of one's true thoughts; internal control of inappropriate expressions of emotion; and self-awareness. Research has demonstrated that leaders who exhibit authentic leadership behavior are perceived as more effective than those who do not (Avolio and Gardner, 2005).

The leader-member exchange (LMX) theory of leadership takes a relationship-based approach in defining leadership. While the other leadership theories outlined above focus exclusively on leaders, LMX theory also considers followers and the nature of the relationships between leaders and followers. The central concept in LMX is that effective leadership processes are the result of mature relationships between leaders and followers who partner to pursue common goals (Graen and Uhl-Bien, 1995).

While a comprehensive definition of leadership remains elusive, the major leadership theories suggest that true leadership talent involves the ability to persuade followers to suspend their purely selfish interests to support and work toward a common good (Hogan and Kaiser, 2005). Boyatzis and McKee (2005) refer to leaders with that kind of talent as resonant leaders, those who have demonstrated that they are able to blend financial, human, intellectual, environmental, and social capital to create positive results and competitive advantage for their organizations.

The literature demonstrates that effective leadership is central to the success of any business, family-controlled or not. In his study of the highly successful turnaround companies featured in *Good to Great*, Collins (2001) discovered that those companies selected a new CEO first, then adopted a winning strategy developed and executed by that CEO and his/her team, rather than the other way around. Collins refers to these highly effective leaders as “Level 5” leaders, who in addition to exhibiting the resonant leadership characteristics identified by Boyatzis and McKee (2005), were modest, humble, and phenomenally persistent. Noted family business expert Ward (1997) emphasizes how important effective leadership is to the sustainable growth of a family enterprise, which often determines a family firm's ability to survive through multiple generations of family ownership. Leadership effectiveness is one of two dependent variables in the study's conceptual model, and the one of primary interest.

### Engagement with work

Work engagement is the positive opposite of burnout and has been identified in studies on positive psychology as a central element of well-being at work (Seppälä et al., 2009). It can be described as “a positive, fulfilling, work-related state of mind that is characterized by vigor, dedication, and absorption” (Schaufeli et al., 2002). Next-generation leaders who are more committed to the business, a key to its long-term survival and success (Miller and Breton-Miller, 2006), are more likely to demonstrate behavior above and beyond what is required by their job description (Dawson et al., 2013), demonstrating a high level of engagement with their work in the family firm. This study explores the effect of shared vision on next-generation engagement with work, our second dependent variable.

### Shared vision

Leadership and family business literature suggests that a true shared vision drives strategy, gives meaning to work, and creates commitment at all levels of an organization (Boyatzis and McKee, 2005; Boyatzis, 2006). It is particularly important in family businesses as without shared vision commitment to continued ownership wanes (Ward, 1988, 2004, 2011; Davis et al., 1997; Poza, 2013). The ability to articulate and inspire commitment to a shared vision is often cited as a key characteristic of effective leaders (Bass, 1985; Goleman et al., 2002; Boyatzis and McKee, 2005; Boyatzis, 2008; Boyatzis and Soler, 2012).

In an earlier quantitative study (Miller, 2014; “Developing next-generation leadership talent in family businesses: Family climate matters”), the author was surprised to find no significant relationship between overall family business climate and next-generation leadership effectiveness. In that study, business climate was assessed using three scales developed by Boyatzis and Akrivou (2006); Boyatzis (2008, 2013) that measure vision, compassion, and overall positive mood. This study is designed to tease out the specific effect of shared vision, excluding the compassion and overall positive mood dimensions of business climate. The following hypotheses follow from the literature's assertions that shared vision is a characteristic of effective leaders and that it gives meaning to one's work:

H1: Having a shared vision for the family business predicts the leadership effectiveness of next-generation family leaders.
H2: Having a shared vision for the family business has a positive effect on the degree to which next-generation family leaders are engaged with their work in the family firm.

### Family climate

Family climate has a strong effect on family business culture and performance (Björnberg and Nicholson, 2007), and is what makes family-owned businesses different from public and non-family privately owned firms. Björnberg and Nicholson (2007) identify three broad categories that define family climate, each of which have two dimensions: (a) family intergenerational style, (b) family cohesion, and (c) family process.

Family intergenerational style refers to the degree of authority exercised by the senior generation and to how much time and attention they devote to the younger generation. In a family business context, it refers to the intergenerational style of all senior family members who exercise authority in the family firm, which may include family members other than parents. An intergenerational style that is over-controlling and oppressive may meet with resistance and rebellion from younger family members (Walsh, 2003) creating conflict that inhibits the development of a shared vision for the family business and the next generation's ability to differentiate themselves and develop leadership skills (Kerr, 1988). On the other hand, an intergenerational style that involves paying adequate attention to the developmental needs of the younger generation fosters healthy family functioning (Björnberg and Nicholson, 2007).

Family cohesion is comprised of cognitive and emotional cohesion. Cognitive cohesion refers to the degree to which family members share worldviews, norms, and values. Cognitive cohesion influences the leadership culture of the family firm and can be used to create competitive advantage through what Habbershon and Williams (1999) identify as the “familiness” of a family enterprise. Emotional cohesion refers to the emotional bonds among family members. Emotional cohesion contributes to positive family relationships, but too much emotional cohesion can become dysfunctional, leading to a family system that is rigid and enmeshed (Beavers and Voeller, 1983). Lack of sufficient cognitive or emotional cohesion often leads to destructive conflicts that put the functioning of the family and the business at risk.

Family process refers to the degree of open communication and adaptability in the family system. Open communication is viewed by family business researchers as a central characteristic of well-functioning family and family business systems (Davis et al., 1997; Ward, 2004; Poza, 2013). Adaptability is critical to the family's ability to make strategic shifts in the business in response to changes in the external environment (Walsh, 2003). Research on conflict style in family firms demonstrates the importance of how families face challenges when working and living together, particularly when those challenges create strain on family relationships (Danes et al., 2000). A family's “conflict style” is influenced by how its members communicate and its receptivity and adaptability to change (Björnberg and Nicholson, 2007).

Björnberg and Nicholson's (2007) components of family climate interact to influence how well the family system functions. Three of the six dimensions; cognitive cohesion, intergenerational authority, and open communication seem most likely to influence the family's ability to develop a shared vision for the family business.

The family business literature is consistent in suggesting a strong link between shared family values and a vision for the family business (Davis et al., 1997; Ward, 1997, 2004, 2011; Poza, 2013). As cognitive cohesion is defined by the degree to which family members share values and norms, it is hypothesized that:

H3: Cognitive cohesion has a positive effect on the degree to which there is a shared vision for the family business.

Senior generation leaders who exercise unquestioned authority create a negative climate that can wreak havoc in an organization (Kets de Vries, 1985), derail the succession process (Morris et al., 1997; Breton-Miller et al., 2004), and make it more difficult to create commitment to the future direction of the business (Kets de Vries, 1994; Björnberg and Nicholson, 2007). In addition, previous research has found that intergenerational authority is orthogonal to the other two family climate scales used in this study (Björnberg and Nicholson, 2007). Consequently, it is hypothesized that:

H4: Intergenerational authority has a negative effect on the degree to which there is a shared vision for the family business.

Family business scholars are consistent in maintaining that open and transparent communication is an essential element of a well-functioning family business system (Davis et al., 1997; Ward, 2004; Poza, 2013). Open and respectful communication builds trust and facilitates decision making, so it is logical to hypothesize that:

H5: Open communication has a positive effect on the degree to which there is a shared vision for the family business.

**Figure 1:**
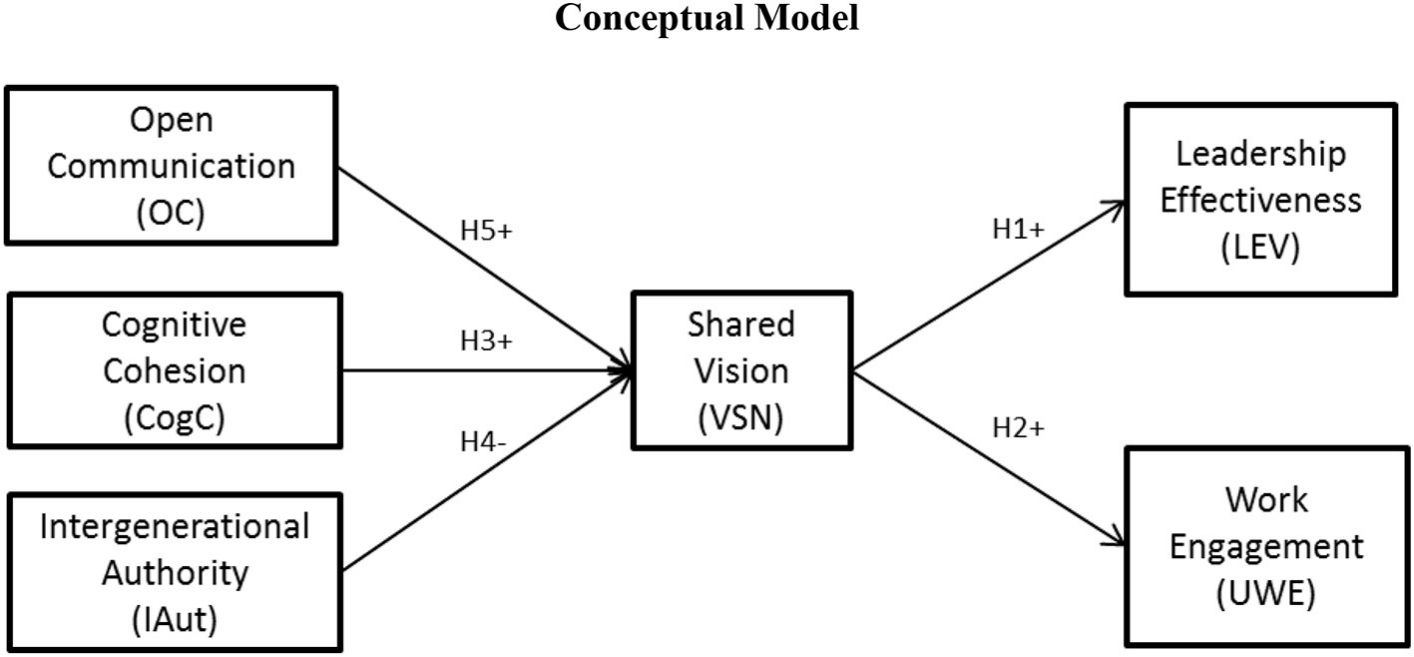
**Conceptual Model**.

The study's theoretical framework and hypothesized relationships are depicted in the conceptual model shown in Figure 1.

## Methods

### Multi-rater cross sectional design

A quantitative survey was designed to capture the perceptions of a cross section of family and non-family members in each family business that participated in the study. Next-generation family business leaders were defined as leaders at any management level who are members of any generation of the business-owning family other than the generation that founded the business. Each next-generation leader who participated filled out a survey and asked three to seven people familiar with his/her leadership practices to fill out a similar survey.

Next-generation leaders in each firm answered questions about the climate of the business-owning family, the degree to which there is a shared vision for the future of the family business, and the nature of their engagement with their work in the family firm. Other family members and non-family members working in the family firm (the “multi-raters”) answered the same set of questions about shared vision and rated the next-generation leader's leadership effectiveness. Multi-raters who were members of the business-owning family also responded to the set of questions about family climate.

The multi-rater, 360° feature is a key element of the study design as it increases the accuracy of results and avoids common method bias. Multi-rater perceptions were used to measure the leadership effectiveness of the next-generation leaders in the study, as self-ratings are often unreliable and inflated (Taylor, 2014). In addition, using different sources to assess key measures is the best *ex ante* procedure to avoid potential common method variance (Podsakoff et al., 2003).

### Measurement development

Scales used to measure each construct in the model and their sources are described below. Five-point Likert-type scales were used as recent research indicates that five-point scales yield higher quality results than seven- or eleven-point scales for agree-disagree rating scales (Revilla et al., 2014). A complete list of survey items is included in the **Appendix**.

### Family climate

Family climate, the nature of family relationships and whole family functioning, was measured using 24 items from three of the Family Climate Scales (Björnberg and Nicholson, 2007). These scales were chosen as they are specifically designed to measure family climate in a family business context. Three dimensions of family climate were measured: (1) Open communication, the degree to which the family openly and frankly communicates; including listening, showing interest in each other's opinions, and dealing forthrightly with issues of concern; (2) Cognitive cohesion, the degree to which family members share norms and values, including attitudes, interests, and beliefs; and (3) Intergenerational authority, the degree to which the senior generation sets the parameters of family conduct, including exercising power, setting the rules, and allowing the younger generation to participate in decision making. In their creation of the scales Björnberg and Nicholson (2007) achieved Cronbach's alphas of 0.85 for open communication, 0.90 for cognitive cohesion, and 0.75 for intergenerational authority. A combined version of the scales was used in Björnberg and Nicholson's (2012) study of next generation emotional ownership in the family firm and achieved a Cronbach's alpha of 0.85. Family climate items were measured with a five-point Likert-type scale ranging from “strongly disagree” to “strongly agree.”

### Family business shared vision

Family business shared vision was measured using eight items from the Positive and Negative Emotional Attractor (PNEA) scale (Boyatzis, 2006, 2008, 2013). Vision is defined as the degree to which management has articulated a clear, inspiring vision for the future of the business that builds on the organization's strengths. The PNEA scale is a relatively new measure that has been used in a number of doctoral dissertations. The shared vision scale achieved Cronbach's alphas of 0.93, 0.91, and 0.86 respectively in three recent doctoral qualifying papers at the Weatherhead School of Management, Case Western Reserve University (Clayton, 2009; Mahon, 2011; Neff, 2011) PNEA items were measured on a five-point Likert-type scale ranging from “strongly disagree” to “strongly agree.”

### Leadership effectiveness

Leadership effectiveness, the extent to which the next-generation leader is perceived to be effective, was measured using five items from the Leadership Effectiveness scale (Denison et al., 1995): (1) Performance standards; (2) Comparison to peers; (3) Performance as a role model; (4) Overall leadership success; and (5) Overall effectiveness as a leader. The scale achieved an alpha of 0.83 in Denison et al. (1995) article on behavioral complexity in managerial leadership. Leadership effectiveness items were measured using a five-point scale with different labeling for the extremes of each item measure.

### Work engagement

Work engagement, a positive, fulfilling, work-related state of mind that is the positive opposite of burnout, was measured using the nine-item version of the Utrecht Work Engagement Scale (Schaufeli and Bakker, 2003). The Utrecht Work Engagement Scale measures three dimensions of work engagement: (1) Vigor, the degree to which the next-generation leader invests energy, effort, and persistence in their work; (2) Dedication, the extent to which the next-generation leader experiences a sense of significance, enthusiasm, inspiration, pride, and challenge in their work; and (3) Absorption, the degree to which the next-generation leader fully concentrates on and becomes deeply engrossed in their work. In an analysis of the construct validity of the nine-item Utrecht Work Engagement Scale using data from five studies (Seppälä et al., 2009), Cronbach's alphas ranged from 0.81 to 0.85 for vigor, 0.83 to 0.87 for dedication, and 0.75 to 0.83 for absorption. Work engagement items were measured using a five-point Likert-type scale ranging from “never” to “consistently.”

### Pre-testing, data collection, and sample

Survey questions were pre-tested using a Q-sort following guidelines suggested by Thomas and Watson (2002). The Q-sort was followed by two pilot tests of the online questionnaire.

Data was collected over a 4-month period from mid-September 2013 to mid- January 2014. Participants were recruited through the primary researcher's personal network of privately-owned family business owners and consultants, university-based family business centers, business trade organizations, and businesses which provide services to family firms.

Approximately 9537 email invitations generated responses from 866 participants for a response rate of 9.1%. Unfinished and incomplete surveys were removed from the database resulting in 567 usable surveys. Because multiple multi-raters were required for each next-generation leader included in the analysis, the data base was further reduced to a matched set of 100 next-generation family leaders and 350 multi-raters for an average of 3.5 multi-raters per next-generation leader. Respondent characteristics are shown in Table 1 and family business characteristics are shown in Table 2.

**Table 1:** **Respondent characteristics**.

	**Matched sample**			
	**NGLs**		**MRs**	
	**Number**	**Percent (%)**	**Number**	**Percent (%)**
Sample size (n)	100		350	
**GENDER**				
Male	81	81	259	74
Female	19	19	88	25
Missing	0	0	3	1
**AGE**				
18–25	1	1	11	3
26–35	28	28	55	16
36–45	23	23	84	24
46–55	31	31	97	28
56–65	17	17	84	24
66+	0	0	16	5
Missing	0	0	3	1
**GENERATION**				
G1	0	0		
G2	41	41		
G3	32	32		
G4	17	17		
G5+	8	8		
Missing	2	2		
**EDUCATION**				
Less than high school	0	0	0	0
High school/GED	2	2	27	8
Some college	6	6	53	15
2-year college degree	2	2	28	8
4-year college degree	58	58	154	44
Master's degree	27	27	77	22
Doctoral degree (PhD, EdD)	2	2	0	0
Professional degree (JD, MD)	3	3	11	3
Missing	0	0	0	0
**POSITION IN FAMILY BUSINESS**				
CEO	51	51	17	5
Other senior-level management	34	34	190	54
Middle-level management	10	10	86	25
Entry-level management	5	5	16	5
Non-management position	0	0	39	11
Missing	0	0	2	1
**FAMILY MEMBERSHIP**				
Family member			61	17
Non-family member			288	82
Missing			1	0
**NGL RELATIONSHIP**				
Immediate supervisor			22	6
Senior leader			36	10
Direct report			144	41
Other follower			45	13
Peer			44	13
Other relationship			51	15
Missing			8	2

**Table 2:** **Family business characteristics**.

**Family business characteristics**	**Matched sample**	
Sample Size (n)	100	
**REVENUE**		
Under $25 million	29	29%
$25–$50 million	9	9%
$51–$100 million	15	15%
$101–$250 million	26	26%
$251–$500 million	9	9%
$500 million+	11	11%
Missing	1	1%
**OWNERSHIP**		
Privately owned	99	99%
Public, but family controlled	0	0%
Public	0	0%
Other form of ownership	1	1%
Missing	0	0%

### Data screening

Total missing data was only 0.1%. Missing values were completely at random and were imputed using the MCMC method in IBM SPSS 22.0.0.0. Several variables exhibited negative skewness and/or kurtosis. Because multivariate analysis assumes normality of data, skewed variables were transformed by squaring or cubing which cured both skewness and kurtosis issues (Hair et al., 2010). All relationships in the model exhibited homoscedasticity and linearity.

### Measurement model analysis

Covariance-based structural equation modeling (CB-SEM) was used to analyze the data. CB-SEM is a widely accepted and powerful regression-based technique for testing causal models with multiple constructs. This method was particularly well suited for this study because it allows modeling of abstract concepts reflective of many indicators (observed variables) such as the six constructs and 34 indicators in the conceptual model. In addition, CB-SEM enables the estimation of causal networks including direct and indirect effects simultaneously (Lowry and Gaskin, 2014), a feature that proved to be important in demonstrating the indirect effects of two of our family climate scales on next-generation leadership effectiveness and engagement with work. IBM AMOS 22.0.0, the most current version of the software at the time of the study, was used to create the measurement model and assess relationships among the constructs.

An exploratory factor analysis (EFA) was conducted first and resulted in a six-factor solution. All indicators loaded cleanly on their respective factors, with values exceeding the 0.50 threshold recommended by Hair et al. (2010) as necessary for practical significance and indicator reliability. The EFA was followed by a confirmatory factor analysis (CFA), which demonstrated good model fit. CMIN/DF was 1.31, less than the maximum threshold of 3.0 recommended by Carmines and McIver (1981). CFI was 0.93, RMSEA was 0.06, and PCLOSE was 0.22, all of which exceed the standards recommended by Hair et al. (2010) for a model with a sample size less than 250 and more than 30 variables. See Table 3 for complete measurement model results.

Table 3**Measurement model results**.

**Table:** 

**Constructs/Items**	**Mean**	**Std. Dev.**	Std. regression weights^*^	**Cronbach's Alpha**	**Composite reliability**	**Average variance extracted**	**Maximum shared variance**
Criteria^**^			>0.50	>0.70	>0.70	>0.50	<AVE
Cognitive cohesion	14.91	4.17		0.86	0.86	0.56	0.50
cog_1_sq	13.13	5.19	0.64				
cog_3_sq	14.49	4.59	0.76				
cog_4_sq	14.34	5.11	0.85				
cog_5_sq	15.69	5.46	0.78				
cog_8_sq	16.91	5.69	0.70				
Intergenerational authority	2.59	0.77		0.82	0.83	0.63	0.22
iaut_3	2.64	0.84	0.70				
iaut_4	2.84	0.97	0.75				
iaut_7	2.30	0.88	0.92				
Leadership effectiveness	14.42	3.58		0.90	0.95	0.80	0.28
lev_1_sq	17.07	4.61	0.90				
lev_2	4.05	0.62	0.85				
lev_3_sq	17.27	5.08	0.89				
lev_4_sq	16.93	4.45	0.90				
lev_5_sq	16.81	4.63	0.94				
Open communication	9.42	2.99		0.81	0.89	0.54	0.50
oc_1	3.56	1.05	0.68				
oc_2_sq	14.84	6.35	0.60				
oc_3	3.33	1.00	0.61				
oc_4_sq	13.23	5.81	0.69				
oc_6_sq	14.11	5.10	0.85				
oc_7_sq	13.38	5.65	0.88				
oc_8	3.50	0.78	0.77				
Work engagement	4.16	0.58		0.88	0.88	0.50	0.13
uwe_1	3.73	0.76	0.74				
uwe_2	3.96	0.78	0.71				
uwe_3	4.33	0.73	0.84				
uwe_4	4.25	0.81	0.79				
uwe_5	4.32	0.78	0.67				
uwe_6	4.35	0.69	0.53				
uwe_8	4.21	0.78	0.65				
Vision	4.06	0.42		0.92	0.92	0.63	0.28
vsn_1	4.10	0.50	0.76				
vsn_2	4.16	0.45	0.75				
vsn_3	4.21	0.48	0.58				
vsn_4	4.03	0.51	0.87				
vsn_6	4.06	0.52	0.88				
vsn_7	3.95	0.58	0.90				
vsn_8	3.90	0.46	0.78

**Table:** 

**MODEL FIT**							
**Statistic**		**Threshold**		**Results**		**References**	
Chi square				657.82			
Degrees of freedom				504			
CMIN/DF		<3.0		1.31		Carmines and McIver, 1981	
CFI		>0.92		0.93		Hair et al., 2010	
RMSEA		<0.07		0.06		Hair et al., 2010	
PCLOSE		>0.05		0.22		Hair et al., 2010	

* p < 0.001 for all standardized regression weights;

** Hair et al. (2010).

Composite reliability and Cronbach's alpha were above the recommended threshold of 0.70 for each of the latent constructs in the model, demonstrating their reliability. Average variance extracted (AVE), which demonstrates convergent validity, was above the recommended threshold of 0.50 (Hair et al., 2010) for all constructs. Tests recommended by Fornell and Larcker (1981) were used to demonstrate the discriminant validly of the constructs. Average variance extracted for each construct was greater than its maximum shared variance (MSV) with any other construct. Discriminant validity was further demonstrated by comparing the square root of AVE for each construct with its highest correlation with any other construct as shown in the correlations matrix in Table 4. In all cases, the square roots of the AVEs were higher than their correlations with any other construct.

**Table 4:** **Correlations matrix**.

	**UWE**	**OC**	**IAut**	**VSN**	**LEV**	**CogC**
UWE	0.71					
OC	0.33	0.73				
IAut	−0.36	−0.28	0.79			
VSN	0.34	0.33	−0.47	0.80		
LEV	0.31	0.30	−0.30	0.53	0.89	
CogC	0.14	0.71	0.00	0.07	0.16	0.75

Square root of AVEs on the diagonals. UWE, engagement with work; OC, open communication; IAut, intergenerational authority; VSN, shared vision; LEV, leadership effectiveness; CogC, cognitive cohesion.

## Results

### Collinearity assessment of predictor variables

Before testing for the significance of path coefficients in the model, the predicator variables were tested for collinearity. Collinearity among predictor variables inflates the standard errors of estimates rendering statistical tests and punctual estimates meaningless. Tolerance and its inverse, the variance inflation factor (VIF), measure collinearity. Tolerance is simply the amount of variance in an independent variable that is not explained by the other independent predictor variables. Tolerance values below 0.20 and VIF values above 5 indicate potential collinearity problems (Hair et al., 2013). IBM SPSS Statistics was used to perform a collinearity analysis on the predictor variables in the model, all of which demonstrated tolerance and VIF values well within acceptable limits (see Table 5).

**Table 5:** **Collinearity assessment**.

**Variable**	**Tolerance**	**VIF**
Cognitive cohesion	0.61	1.64
Intergenerational authority	0.76	1.31
Open communication	0.54	1.85
Shared vision	0.74	1.36

### Coefficients of determination

**Figure 2:**
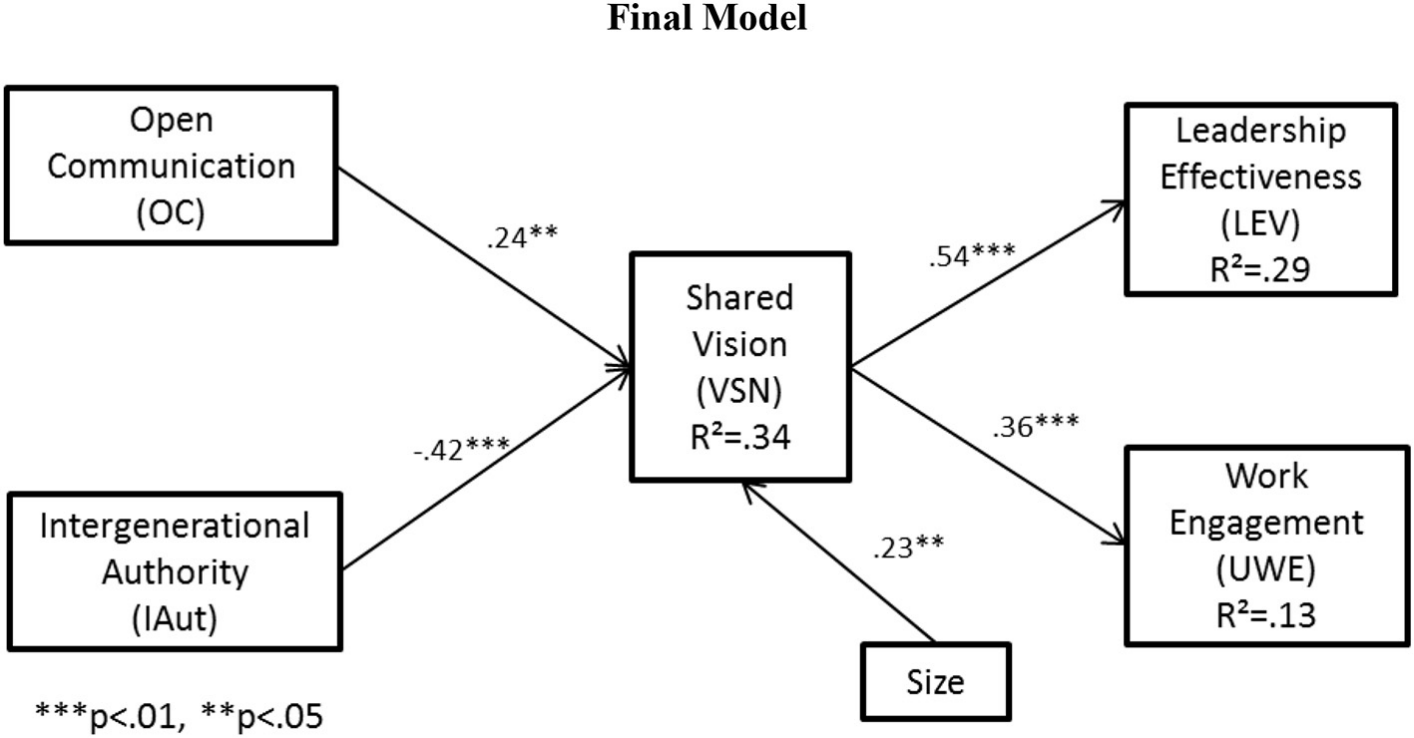
**Final Model**.

Coefficients of determination (R^2^) values for each of the three endogenous latent constructs in the model are shown in the final model in Figure 2. R^2^ values measure the amount of variance in the construct explained by the exogenous variables in the model. R^2^ was 0.34 for vision, 0.29 for next-generation leadership effectiveness, and 0.13 for next-generation work engagement. While there are no universal standards for acceptable R^2^ values, these values of are practical significance for the purposes of this study (Hair et al., 2013).

### Significance of path coefficients

The size and significance of the path coefficients of hypothesized relationships in the structural equation model were determined by calculating estimates in AMOS. Results are summarized in Table 6 and discussed for each hypothesized relationship below.

**Table 6:** **Significance testing results of structural equation model path coefficients**.

**Path**	**Standardized coefficient**	**Standard error**	**Critical ratio**	***p*-value**
CogC -> VSN	−0.20	0.02	−1.27	0.203
IAut -> VSN	−0.37	0.05	−3.26	0.001
OC -> VSN	0.40	0.11	2.34	0.019
VSN -> LEV	0.53	1.14	5.06	^***^
VSN -> UWE	0.36	0.17	3.19	0.001
Size -> VSN	0.24	0.02	2.66	0.008

*** p < 0.001. UWE, engagement with work; OC, open communication; IAut, intergenerational authority; VSN, shared vision; LEV, leadership effectiveness; CogC, cognitive cohesion.

### Effect size f^2^

An additional step in evaluating the predictive power of a structural equation model is to calculate the relative contribution of each exogenous variable in the model to the coefficient of determination (R^2^ value) of the endogenous variable it predicts. The formula for calculating f^2^ values is as follows:

$${\text{f}}^{\;2}=\frac{{\text{R}}^{2}\text{included}-{\text{R}}^{2}\text{excluded}}{1-{\text{R}}^{2}\text{included}},$$

where R^2^ included and R^2^ excluded are the R^2^ values of an endogenous latent variable when a selected exogenous latent variable is included in or excluded from the model (Hair et al., 2013). Cohen (1988) suggests that f^2^ values of 0.02, 0.15, and 0.35 respectively, represent small, medium, and large effects. Results of tests for f^2^ effect sizes are displayed in Table 7 and discussed for each hypothesized relationship below.

**Table 7:** **f^2^ Effects**.

**Path**	**Standardized coefficient**	**f^2^ Effect value**	f^2^ Effect size^*^
IAut -> VSN	−0.42	0.24	Medium
OC -> VSN	0.24	0.06	Small
VSN -> LEV	0.54	0.41	Large
VSN -> UWE	0.36	0.15	Medium

* (Cohen, 1988), UWE, engagement with work; OC, open communication; IAut, intergenerational authority; VSN, shared vision; LEV, leadership effectiveness.

### Results of hypothesis testing

*H1: Having a shared vision for the family business predicts the leadership effectiveness of next-generation family leaders.* As expected, shared vision for the family business strongly predicted the leadership effectiveness of the next-generational family leaders in our study (0.53, *p* < 0.001), supporting H1. The f^2^ effect (0.41) was large, demonstrating the importance of shared vision in determining the variance in next-generation leadership effectiveness in the model. As 51% of the next-generation leaders in our study were CEOs and another 34% held other senior leadership positions, this result underscores the degree to which the ability to articulate and communicate a shared organizational vision is associated with the perceived leadership effectiveness of senior leaders. What is particularly important in this study is the indirect influence of family climate on next-generation leader effectiveness through shared vision, examined in greater detail in the Discussion section of the paper.

*H2: Having a shared vision for the family business has a positive effect on the degree to which next-generation family leaders are engaged with their work in the family firm*. H2 was also supported as shared vision demonstrated a meaningful effect (0.36, *p* < 0.01) on the degree to which next-generation leaders reported positive engagement with their work. The f^2^ effect (0.15) was medium. This result demonstrates that having a shared vision for the family business contributes to the sense of fulfillment, energy, and enthusiasm experienced by the next-generation leaders in our study.

*H3: Cognitive cohesion has a positive effect on the degree to which there is a shared vision for the family business.* The big surprise in the results was that cognitive cohesion had no effect (−0.20, *p* > 0.10) on the degree to which the family firms in our study had shared visions. This result contradicts the link between shared family values and a shared vision for the family business almost universally suggested in the family business literature. Because no effect was an unexpected result, the statistical power of the model for shared vision was calculated and found to be 0.99, well above the recommended minimum threshold of 0.80 (Ellis, 2010), thus providing confidence in the result. Possible explanations for this unexpected finding are advanced in the Discussion section below.

*H4: Intergenerational authority has a negative effect on the degree to which there is a shared vision for the family business.* H4 was supported as intergenerational authority had a substantial negative effect (−0.37, *p* < 0.01) on the degree to which the family firms in the study had shared visions. The f^2^ effect (0.24) was medium. This finding is of particular importance as it suggests that entrepreneurs who have employed an authoritarian, take-charge leadership style in overcoming the challenges of founding and growing a successful business may find that the same leadership behaviors work against them in preparing the business and the family for a smooth transition to the next generation.

*H5: Open communication has a positive effect on the degree to which there is a shared vision for the family business.* H5 was also supported as open communication demonstrated a significant positive effect (0.40, *p* < 0.05) on shared vision. The f^2^ effect (0.06) was small, which is meaningful although not as strong as expected. The results suggest that open and transparent communication in the family facilitates the development and adoption of a shared vision for the family firm, confirming what is often suggested by family business experts as a fundamental characteristic of family firms that survive through multiple generations of family ownership.

A summary of hypothesis test results is provided in Table 8. The structural equation model was trimmed of the insignificant effect of cognitive cohesion on shared vision following hypothesis testing to produce the final model shown in Figure 2. Final path coefficient estimates and significance levels are shown in Figure 2 and Table 9.

**Table 8:** **Summary of hypothesis test results**.

**Hypothesis**	**Coefficient**	**Support for hypothesis**
*H1: Having a shared vision for the family business predicts the leadership effectiveness of next-generation family leaders.*	0.53^***^	Yes
*H2: Having a shared vision for the family business has a positive effect on the degree to which next-generation family leaders are engaged with their work in the family firm.*	0.36^***^	Yes
*H3: Cognitive cohesion has a positive effect on the degree to which there is a shared vision for the family business.*	−0.20 (ns)	No
*H4: Intergenerational authority has a negative effect on the degree to which there is a shared vision for the family business.*	−0.37^***^	Yes
*H5: Open communication has a positive effect on the degree to which there is a shared vision for the family business.*	0.40^***^	Yes

*** p < 0.01, ns, non-significant.

**Table 9:** **Significance testing results of structural equation model total effects**.

**Path**	**Standardized coefficient**	***p*-value**
IAut -> LEV	−0.22	0.004
IAut -> UWE	−0.15	0.006
IAut -> VSN	−0.42	0.004
OC -> LEV	0.13	0.033
OC -> UWE	0.09	0.037
OC -> VSN	0.24	0.033
Size -> LEV	0.12	0.008
Size -> UWE	0.08	0.012
Size -> VSN	0.23	0.008
VSN -> LEV	0.54	0.004
VSN -> UWE	0.36	0.007

UWE, engagement with work; OC, open communication; IAut, intergenerational authority; VSN, shared vision; LEV, leadership effectiveness.

### Total effects

The total effects (direct and indirect) of exogenous variables on endogenous variables provide the greatest insight (Hair et al., 2013). The structural equation model test results demonstrated that there were significant positive indirect effects of open communication on next-generation leadership effectiveness (0.13, *p* < 0.05) and engagement with work (0.09, *p* < 0.05) through the mediating variable shared vision. There were also indirect negative effects of intergenerational authority on leadership effectiveness (−0.22, *p* < 0.01) and engagement with work (−0.15, *p* < 0.01), also through shared vision. These are two of the more important findings in the study as they demonstrate that family climate has meaningful effects on two of the variables most closely associated with multi-generational family business success, shared vision and capable next-generation leadership. Total effects for all significant relationships in the model are reported in Table 9.

### Controls

Size of family business, as measured by revenue, and age of next-generation leader were included as controls. Size was significantly related to shared vision (0.23, *p* < 0.01) but not with any of the other variables in the model. Age had no significant relationships with any of the variables in the model.

## Discussion

The study explored the relationships among shared vision, family climate, and next-generation leadership effectiveness and engagement with work; key factors associated with the multi-generational success of family-owned enterprises. Interpretations of the major findings, including implications for practice, suggestions for future research, and limitations of the study follow.

### Shared vision predicts next-generation leadership effectiveness

The results demonstrate that the presence of a shared vision for the family business strongly predicts the effectiveness of next-generation family leaders. There are two important implications of this finding. As most of the next-generation leaders in the study were CEOs or other members of senior management, it suggests that next-generation family leaders who are skilled at creating and articulating a shared vision for the family firm are more likely to be perceived as effective leaders by family and non-family members working in the business. It also suggests that business-owning families who take the time to do the hard work of creating a shared vision for the family business increase the chances of developing next-generation family leaders who exhibit effective leadership behaviors. It is reasonable to infer that if senior generation family members model cooperative behavior in creating a shared vision for the family business, next-generation family members are more likely to value and learn that skill, which in turn affects their own leadership behavior and effectiveness. So it turns out that two of the top three factors associated with family business longevity (Ward, 1997) are strongly related and can be simultaneously addressed by creating a shared vision for the family business.

This finding provides some insight into the surprising results of an earlier study that showed no effect of a comprehensive measure of business climate that included shared vision, compassion, and overall positive mood on next-generation leadership effectiveness (Miller, 2014). That more comprehensive measure has been shown to be related to leadership effectiveness in other contexts (Boyatzis and McKee, 2005). Perhaps in a family business, the presence of compassion and overall positive mood in the business are attributed to the business-owning family or founder of the family business, rather than next-generation leaders. While the results of this study partially resolve the conundrum of the earlier finding, further exploration in future studies of family businesses and their leaders is warranted.

### Shared vision positively affects next-generation leader engagement with work

Although not as strong as the relationship with leadership effectiveness, shared vision also had a meaningful effect on the degree to which next-generation leaders reported themselves to be fulfilled and energized by their work in the family firm. This was the expected result as leadership literature is consistent in its assertion that shared vision creates commitment and provides meaning and purpose to one's work in an organization (Kantabutra, 1992; Boyatzis and McKee, 2005; O'Connell et al., 2011). It may be even more important in a family business context as socio-emotional goals are often more important than financial goals (Gómez-Mejía et al., 2007).

This suggests that business-owning families would be wise to create a clear vision for the business that is meaningful to next-generation family leaders if they want to encourage them to pursue careers in the family enterprise. It further suggests that next-generation family leaders have some control over their own destiny in the family firm. If they work with other family members to create a shared vision for the business that is also personally inspiring, they are more likely to experience a fulfilling career. On the other hand, if this is not possible, they may find a career outside of the family firm to be more rewarding.

### Family climate affects shared vision for the family business

While shared vision has been shown to be important to organizational outcomes in other contexts, this study makes an important contribution to the literature by demonstrating how the climate of the business-owning family affects the degree to which a shared vision is present in a family-owned enterprise, and as a result, the leadership effectiveness and work engagement of next-generation family leaders. The effects of open communication, intergenerational authority, and cognitive cohesion, three dimensions of family climate hypothesized to be important to the presence of a shared vision were examined.

Open communication is often viewed as the sine qua non of effective family businesses (Davis et al., 1997; Ward, 2004; Carlock and Ward, 2010). This study confirmed its importance in creating a shared vision for the family firm. That is hardly surprising as it is difficult to imagine how a business-owning family could create a shared vision without communicating. Nonetheless, the history of family business is punctuated by highly publicized examples of family firm blow-ups characterized by poor communication among family members (Poza, 2013). This study provides insight into how that can happen, by demonstrating the strong negative effect of intergenerational authority on shared vision. A senior generation that exercises unquestioned authority and makes all the rules puts the firm at significant risk by diminishing the chances that family owners will be able to create a vision for the business to which all are committed. This is quite important as the fiercely independent authoritative leadership style that is common among many entrepreneurs (De Vries, 1977; Kets de Vries, 1985) and may have helped them overcome the enormous challenges of founding or growing a successful business may work against them in preparing the family firm for transition to future generations of family ownership.

The results also show that the positive effect of open communication and negative effect of intergenerational authority extend to next-generation family leaders. Open communication indirectly affects next-generation leader effectiveness (0.13, *p* < 0.05) and engagement with work (0.09, *p* < 0.05) through its effect on vision. Intergenerational authority indirectly affects next-generation leadership effectiveness (−0.22, *p* < 0.01) and engagement with work (−0.15, *p* < 0.01) through its effect on vision. So the nature of the family climate has meaningful effects on both vision and next-generation family leadership, two of the most important predictors of multi-generational family business success.

The surprise in the findings was that there was no effect of cognitive cohesion on shared vision. The family business literature is virtually universal in asserting that shared values among family members (Davis et al., 1997; Ward, 2004, 2011; Carlock and Ward, 2010; Boyatzis and Soler, 2012; Poza, 2013), a strong indicator of cognitive cohesion in the study (0.70, *p* < 0.001), are important to creating a vision for the family firm. One interpretation of this result is that it may be possible for family members to have different personal values and views on issues but still be able to coalesce around a shared vision for the family business. Because open communication had a positive effect on shared vision, perhaps family owners can set aside personal differences to create a shared vision for the family firm if they have effective ways of communicating. Another plausible explanation is that the family members in our study do not view the family business as the vehicle through which personal or family values should be expressed, choosing instead to view family and business as separate domains.

It is also possible that emotional cohesion in the family, which was not included as a construct in the study, is more important than cognitive cohesion in developing a shared vision for the family business. Murray (2002) points out that the marriage of the rational and the emotional is a unique characteristic of family businesses. In their work on family climate, Björnberg Nicholson assert that “emotional ownership” is important to the development of a shared vision for the family business (Björnberg and Nicholson, 2012) and to the commitment of next-generation family members to the family enterprise. While this study's finding of a lack of effect of cognitive cohesion on shared vision is important, additional research to test the effects of cognitive as well as emotional cohesion on shared vision in family firms is suggested.

What seems clear for practitioners is that creating processes like regular family meetings to facilitate open communication among family members may enhance the chances of multi-generational survival for family firms through positive effects on shared vision and next-generation leadership. The message for the successful senior-generation family entrepreneur is that learning and practicing communication skills with family members may make it more likely that the business they have worked so hard to create will continue to prosper beyond their own tenure as leader.

### The size of the family business is related to the presence of a shared vision

Although included as a control variable, size of the family business turned out to have a meaningful relationship with the presence of a shared vision (0.23, *p* < 0.01). It may be that larger family firms simply have more structures and processes in place to create and communicate a clear vision and strategy for the business. However, the more important implication is that family firms that develop a shared vision are more likely to grow, thus providing support for the widely held tenet that shared vision is important to long-term family business success.

### Limitations

Next-generation leaders represented in the study were self-selected since they voluntarily responded to email invitations to participate, thus they did not comprise a strictly random sample. This introduces the threat of external validity, the ability to generalize results across all family businesses (Shadish et al., 2002), so the results should be viewed with the possibility of that limitation in mind. Next-generation leaders also nominated their own multi-raters introducing the possibility of social desirability in multi-rater responses, a potential threat to construct validity, the ability to generalize causes and effects (Shadish et al., 2002), although this is a risk inherent to most 360° leadership surveys. Nonetheless, there was sufficient variation in multi-rater evaluations to provide confidence in the reliability of our results. As with most studies of this nature, the constructs were measured at a specific point in time. Concepts like shared vision and leadership effectiveness develop over time, so theoretically a longitudinal study would be ideal. However, there is enough variation in the age of the next-generation leaders who participated and in the generational stage of ownership of their family firms to provide confidence in the reliability of the findings.

## Conclusion

The study demonstrates that a shared vision for the family business strongly predicts the leadership effectiveness of next-generation family leaders and affects the degree to which they are positively engaged with their work in the family firm. The findings also show that the climate of the business-owning family significantly influences the creation of a shared vision for the family firm, and as a result, the development of next-generation leadership talent. Thus, three of the factors most closely associated with multigenerational family business longevity are meaningfully related. The implications for family business owners is that they would be wise to spend as much time fostering a positive family climate as they do on creating a successful business strategy if their goal is to pass the business from one generation of family owners to the next.

### Conflict of interest statement

The author declares that the research was conducted in the absence of any commercial or financial relationships that could be construed as a potential conflict of interest.

## Appendix

### Survey items

**Table:**

**Item code**	
	**FAMILY CLIMATE—OPEN COMMUNICATION**
oc_1	People don't openly express their opinions (RC)
oc_2	We keep our views pretty much to ourselves (RC)
oc_3	We are polite rather than honest in how we communicate with each other (RC)
oc_4	We regularly talk about things that concern us
oc_5	People are not interested in each other's opinions (RC)
oc_6	We take time to listen to each other
oc_7	We bring issues out in the open, good or bad
oc_8	We are frank with each other
	**FAMILY CLIMATE—COGNITIVE COHESION**
cogc_1	We have similar views on things
cogc_2	We tend to have widely differing views on most social issues (RC)
cogc_3	We have shared interests and tastes
cogc_4	Our attitudes and beliefs are pretty similar
cogc_5	We do not have much in common (RC)
cogc_6	We think alike
cogc_7	We have radically different perspectives on things (RC)
cogc_8	Our values are very similar
	**FAMILY CLIMATE—INTERGENERATIONAL AUTHORITY**
iaut_1	The younger generations try to conform with what older generation would want
iaut_2	The wishes of the older generation are obeyed
iaut_3	The authority of the older generation is not questioned
iaut_4	Family members of the older generation set the rules
iaut_5	We make decisions with every person having an equal say, regardless of seniority (RC)
iaut_6	Older and younger members have equal amounts of power (RC)
iaut_7	The word of the older generation is law
iaut_8	Younger generation is encouraged to freely challenge opinions of older generation (RC)
	**FAMILY BUSINESS VISION**
vsn_1	Management emphasizes a vision for the future
vsn_2	We often discuss possibilities for the future
vsn_3	Our future as an organization will be better than our past
vsn_4	I feel inspired by our vision and mission
vsn_5	We are encouraged by management to and build on our strengths
vsn_6	Our work is focused on our vision or mission
vsn_7	Our purpose as an organization is clear in our vision or mission
vsn_8	Management emphasizes our current strengths
	**LEADERSHIP EFFECTIVENESS**
lev_1	Meets leadership performance standards
lev_2	Comparison to leadership peers
lev_3	Performance as a role model
lev_4	Overall leadership success
lev_5	Overall effectiveness as a leader
	**UTRECHT WORK ENGAGEMENT**
uwe_1	At my work, I feel that I am bursting with energy
uwe_2	At my job, I feel strong and vigorous
uwe_3	I am enthusiastic about my job
uwe_4	My job inspires me
uwe_5	When I get up in the morning, I feel like going to work
uwe_6	I feel happy when I am working intensely
uwe_7	I am proud of the work that I do
uwe_8	I am immersed in my work
uwe_9	I get carried away when I'm working
